# Thoracic vertebral CT-based bone health indicator from coronary calcium scoring: correlation with CAD and prognostic impact

**DOI:** 10.1186/s12872-026-06284-9

**Published:** 2026-07-16

**Authors:** Naoki Onoda, Satoshi Nakamura, Naoki Hashimoto, Suguru Araki, Hajime Sakuma, Kakuya Kitagawa

**Affiliations:** 1 Department of Radiology, Mie University Hospital, 2-174 Edobashi, Tsu, Mie 514-8507 Japan; 2 Department of Advanced Diagnostic Imaging, Mie University Graduate School of Medicine, 2-174 Edobashi, Tsu, Mie 514-8507 Japan; 3 MUDX Initiative, Mie University Organization for Research Initiative and Promotion, 1557 Kurimamachiyacho, Tsu, Mie 514-8507 Japan

**Keywords:** Osteoporosis, Coronary artery calcium, Computed tomography, Mortality, Risk stratification

## Abstract

**Background:**

Lower thoracic vertebral attenuation measured on coronary artery calcium scoring images may provide an opportunistic computed tomography-based bone health indicator (CT-BHI). This study evaluated the association of CT-BHI with coronary artery disease (CAD) severity and all-cause mortality.

**Methods:**

We retrospectively analyzed 767 patients with suspected CAD who underwent coronary CT angiography (CCTA) and coronary calcium scoring. Individuals with prior myocardial infarction or revascularization were excluded. CT attenuation was measured in two lower thoracic vertebrae on calcium scoring images, and their mean value was defined as CT-BHI. Patients were categorized into tertiles by CT-BHI (higher, intermediate, lower).

**Results:**

The lower CT-BHI group had significantly higher calcium scores (median [IQR]: 146 [4–548]) than the higher (25 [0–277]) and intermediate (39 [0–269]) groups (*p* < 0.001). CCTA findings revealed a significant difference in CAD severity distribution among the three groups (*p* = 0.037), with the low CT-BHI group exhibiting a lower prevalence of normal coronary arteries. Combining low CT-BHI with high calcium score enhanced risk stratification (*p* < 0.05). In multivariate Cox analysis, CT-BHI remained an independent predictor of mortality after adjustment for calcium score (*p* < 0.05). Adding CT-BHI to calcium scores significantly improved risk classification (continuous net reclassification improvement, 0.263; *p* = 0.041).

**Conclusions:**

CT-BHI derived from routine calcium scoring images may provide a dual-purpose opportunistic marker for integrated cardiovascular and skeletal risk assessment without additional radiation exposure or cost. Further studies are warranted to validate CT-BHI against standardized bone density measurements and determine its role in improving clinical management.

**Clinical trial number:**

Not applicable.

**Supplementary Information:**

The online version contains supplementary material available at https://doi.org/10.1186/s12872-026-06284-9.

## Background

Osteoporosis and coronary artery disease (CAD) are age-related diseases that represent major public health challenges due to their high prevalence and associated morbidity and mortality [1, 2]. It is well established that osteoporosis is associated with an increased risk of future cardiovascular events, such as myocardial infarction, and higher all-cause mortality [3, 4]. Because coronary atherosclerosis underlies most cardiovascular events, it is clinically important to clarify whether osteoporosis is directly related to the presence and severity of coronary atherosclerosis and calcification. However, the relationship between osteoporosis and the presence or severity of coronary atherosclerosis and calcification remains a subject of debate, with inconsistent findings across studies [5–9]. Moreover, for patients undergoing evaluation for CAD, it is unknown whether assessing bone mineral density (BMD) can provide additive prognostic value for long-term outcomes beyond that obtained from CAD evaluation. Therefore, it remains uncertain whether concurrent screening for osteoporosis is clinically warranted in patients undergoing evaluation for suspected CAD.

The diagnostic standard for osteoporosis is dual-energy X-ray absorptiometry (DXA), while coronary atherosclerosis can be non-invasively quantified using the coronary artery calcium (CAC) score derived from non-contrast computed tomography (CT). Recently, there has been growing interest in using CT attenuation of thoracic vertebrae, obtained from the CAC scans, as a CT-based bone health indicator (CT-BHI) [5]. This opportunistic screening approach is advantageous as it allows for the assessment of bone status without additional radiation exposure or cost. Previous work showed that low thoracic BMD derived from cardiac CT was associated with a greater rate of fractures [6]. As CT-BHI is obtained simultaneously with the coronary artery calcium score (CACS), it is particularly well-suited for investigating its correlation with the presence and severity of CAD and for assessing its additive prognostic value in patients with suspected CAD.

Accordingly, the purposes of the current study were as follows: to investigate the relationship between CT-BHI, as measured on CAC scoring images, and the presence and severity of CAD; to determine whether CT-BHI offers independent and additive prognostic value for all-cause mortality beyond that provided by the CAC scoring. We hypothesized that lower CT‑BHI would be associated with the presence of CAD and would improve prognostic performance when added to CACS.

## Methods

### Study population

We retrospectively identified patients who met all of the following inclusion criteria: 1) evaluation for CAD; and 2) undergoing both CAC scoring and coronary CT angiography (CCTA) at our hospital between April 2012 and March 2019. A total of 1904 patients met these inclusion criteria. Exclusion criteria were non-ischemic cardiomyopathy (*n* = 62); history of myocardial infarction (*n* = 263); history of coronary revascularization (percutaneous coronary intervention or coronary artery bypass grafting) (*n* = 206); severe valve disease (*n* = 238); aortic disease (*n* = 128); congenital heart disease (*n* = 185); cardiac tumors (*n* = 9); and CAC scans unsuitable for CT-BHI assessment (*n* = 46) including lower thoracic vertebrae outside the field of view (*n* = 44), chronic vertebral compression fracture (*n* = 1), and severe scoliosis (*n* = 1). There were no patients with severe degenerative changes in the lower thoracic vertebrae. The final cohort comprised 767 patients after applying these exclusion criteria (Supplemental Fig. 1). The institutional review board in our hospital approved the protocols for this retrospective study and waived the need to obtain individual consent based on the retrospective design.

### Image acquisition

All CT data were acquired with a second-generation (SOMATOM Definition Flash, Siemens Healthineers, Forchheim, Germany) or third-generation dual-source CT scanner (SOMATOM Force, Siemens Healthineers). After anteroposterior and lateral topograms, unenhanced CT images for coronary artery calcium scoring were acquired using prospectively Electrocardiographic (ECG)-triggered high-pitch spiral scan with a tube voltage of 120 kV. Tube current was determined using the automatic exposure control system with a reference tube current–time product of 80 mAs for the second-generation scanner or 50 mAs for the third-generation scanner. These images were reconstructed with a slice thickness and increment of 3 mm using a medium smooth convolution kernel (I30 for the second-generation scanner or Qr36 for the third-generation scanner).

Subsequently, standard prospective CCTA was performed at rest by bolus injection of 26mgI/kg/sec of iopamidol over 12 s with the coronary arteries dilated with a nitrate. Heart rate was controlled before CCTA with intravenous injection of landiolol hydrochloride (Corebeta, Ono Pharmaceutical Co., Ltd, Osaka, Japan), if necessary. The acquisition parameters of CCTA in the 2nd or 3rd-generation scanner were as follows: collimation = 128 × 0.6 or 192 × 0.6 mm, rotation time = 0.28 or 0.25 s, and tube voltage of either 70 or 80 kVp was manually selected based on patient body mass index, respectively. Tube current was determined using the automatic exposure control system with the quality reference of 350 or 300 mAs/rotation at 120 kV. Axial images of CCTA were reconstructed with the smooth kernel (I26 or Br40) and iterative reconstruction technique (strength 3, SAFIRE or ADMIRE, Siemens). Reconstructed thickness and increments were 0.6 mm and 0.3 mm, respectively.

### CACS and CCTA analysis

CAC was quantified from unenhanced CT scans using the Agatston scoring method. Assessment of CCTA was visually performed based on a consensus between two radiologists (S.N. and K.K.) with over 10 years of experience in cardiac CT. CCTA images were visually assessed for stenosis in coronary segments with a reference diameter ≥ 1.5 mm. The severity of stenosis was classified using the Coronary Artery Disease Reporting and Data System (CAD-RADS) as follows: 0: no atherosclerotic change; 1: 1–24% stenosis; 2: 25–49% stenosis; 3: 50–69% stenosis; 4 A: 70–99% stenosis in 1 to 2 vessels; 4B: 70–99% stenosis in 3 vessels or ≥ 50% stenosis in the left main coronary artery; and 5: 100% [10]. Obstructive CAD was defined as having at least one coronary artery with ≥ 50% luminal narrowing on CCTA.

### CT-BHI measurement on CAC images

The target vertebrae were estimated to be T10 and T11. The T10/11 intervertebral disc was identified as the disc closest to the axial plane containing the superior border of the right lung-liver interface. The reliability of this landmark-based approach was confirmed in a separate validation analysis, in which the estimated vertebral level showed high concordance with the actual level determined by whole-spine tracing (Supplementary Material 1).

**Fig. 1:**
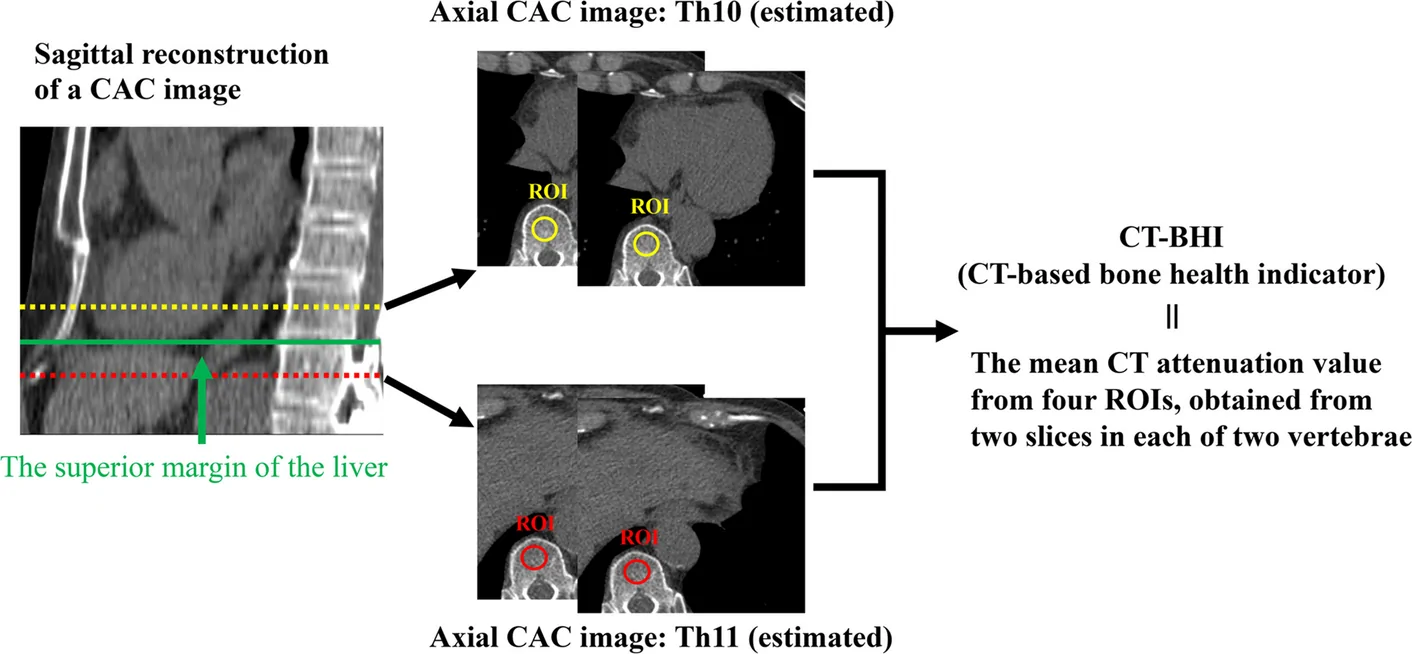
Measurement of CT-BHI on CAC images. The superior aspect of the right lung-liver interface was used as an anatomical landmark to estimate the T10/11 intervertebral disc. Based on this landmark, the estimated T10 and T11 vertebral bodies were identified on axial CAC images. Circular ROIs were placed within the central trabecular bone on two consecutive mid-vertebral axial slices for each vertebral body, avoiding cortical bone, superior and inferior endplates, the venous plexus, focal calcifications, and sclerotic lesions. The mean CT attenuation value from four ROIs was defined as CT-BHI. CAC, coronary artery calcium; CT-BHI, computed tomography-based bone health indicator; HU, Hounsfield unit; ROI, region of interest

CT-BHI was measured on ECG-gated, non-contrast CAC scoring images acquired at 120 kV. All CT-BHI measurements, including vertebral level identification, slice selection, and region of interest (ROI) placement, were performed by a radiologist (N.O.) with 3 years of experience in diagnostic radiology. For each of the estimated T10 and T11 vertebrae, two consecutive axial slices at the mid-vertebral body level were selected while avoiding the superior and inferior endplates. Slice selection was primarily performed on axial CAC images, with reference to adjacent slices to ensure that the ROIs were placed at the mid-vertebral level rather than near the endplates. A circular ROI of approximately 100–150 mm^2^ was placed in the central trabecular bone of each vertebral body. Care was taken to avoid cortical bone, the venous plexus, focal calcifications, and sclerotic lesions. When mild degenerative changes were present, the ROI was placed away from endplate-adjacent or marginal degenerative changes and confined to visually preserved central trabecular bone. The mean CT attenuation value from four ROIs, obtained from two slices in each of two vertebrae, was defined as CT-BHI (Fig. 1). Patients were subsequently stratified into three groups according to CT-BHI tertiles, using cutoff values of 120 and 163 HU.

### Follow-up and endpoint definition

Follow-up information was gathered through a review of hospital records. The primary endpoint was all-cause death.

### Statistical analysis

Continuous variables were expressed as mean ± standard deviation (SD) or median (interquartile range [IQR]), and were assessed using the Student’s t-test or one-way analysis of variance, as appropriate. Categorical variables were expressed as frequencies (percentages) and were compared using Chi-square test. In the DXA validation subset, the associations between CT-BHI and DXA-derived indices were assessed using Spearman correlation analyses and linear regression models. Comparisons of DXA-derived indices across CT-BHI tertiles were performed using the Kruskal–Wallis test. Event-free survival curves were generated using the Kaplan–Meier method and compared using the log-rank test. To identify predictors of the primary endpoint, univariable and multivariable Cox proportional hazards regression analyses were performed. Results are reported as hazard ratios (HRs) with 95% confidence intervals (CIs). Variables with a *p*-value < 0.05 in the univariable analysis were entered into a multivariable model using a “force” method to identify independent predictors. To assess the incremental predictive value of CT-BHI beyond CACS, we calculated the category-free Net Reclassification Improvement (continuous NRI) and the Integrated Discrimination Improvement (IDI). CT-BHI tertiles were used for descriptive analyses of baseline characteristics, imaging findings, and supplemental DXA validation analyses. The tertile cutoff values were 120 and 163 HU. For survival analyses, receiver operating characteristic (ROC) curve analysis for all-cause mortality was performed to determine exploratory optimal cutoff values for CT-BHI and CACS using the Youden index. The ROC-derived cutoff values were used for Kaplan–Meier survival analyses and Cox proportional hazards regression models. The 95% confidence intervals for the CT-BHI and CACS cutoffs were estimated using bootstrap resampling. To address the potential confounding effect of renal function, additional sensitivity analyses were performed using estimated glomerular filtration rate (eGFR) and serum creatinine. eGFR was calculated from serum creatinine, age, and sex using the equation recommended for Japanese individuals: eGFR (mL/min/1.73 m^2^) = 194 × serum creatinine^−1.094^ × age^−0.287^ (× 0.739, if female). To determine whether renal function influenced the prognostic value of CT-BHI, eGFR and creatinine were separately added to the Cox proportional hazards models. A two-sided *p*-value < 0.05 was considered statistically significant. All statistical analyses were conducted using the R statistical package (version 3.4.4, R Foundation for Statistical Computing, Vienna, Austria), and a *p*-value of less than 0.05 was considered statistically significant.

## Results

### Patient characteristics

Baseline characteristics of the 767 study participants are summarized in Table 1. The mean age was 66 ± 12 years, and 462 patients (60%) were male. Hypertension was the most common cardiovascular risk factor, followed by smoking, dyslipidemia, and diabetes. Medication use is also shown in Table 1.

**Table 1:** Patient characteristics

Characteristic	All patients (*n* = 767)
Age, year (mean ± SD)	66 ± 12
Male	462 (60)
Body mass index (mean ± SD)	22 ± 5
Hypertension	482 (63)
Diabetes	212 (28)
Dyslipidemia	337 (44)
Smoking	344 (45)
Family history of CAD	132 (17)
Chest pain	400 (52)
Medication	
ACEI/ARB	279 (37)
Beta-blocker	69 (9)
Antiplatelet agent	167 (22)
Statin	225 (29)
Insulin	48 (6)
Oral antidiabetic agent	109 (14)

Values are n (%), if not indicated otherwise

*ACEI/ARB* angiotensin converting enzyme inhibitors/angiotensin-receptor blockers, *CAD* coronary artery disease, *SD* standard deviation

### Imaging results

The median CT-BHI in the overall cohort was 140 HU (IQR, 110–178). Imaging findings stratified by CT-BHI tertiles are summarized in Table 2. Lower CT-BHI was associated with a greater burden of coronary calcification. The median CACS was highest in the lower CT-BHI group and significantly lower in the intermediate and higher CT-BHI groups (*p* < 0.001). Similar trends were observed when CACS was analyzed categorically and when using the cutoff of CACS ≥ 140. CCTA findings also differed across CT-BHI tertiles. The distribution of CAD-RADS categories was significantly different among the three groups (*p* = 0.037), with the lower CT-BHI group showing a lower prevalence of CAD-RADS 0 and a higher proportion of coronary atherosclerotic disease.

**Table 2:** Imaging results

Parameters	All patients (*n* = 767)	Lower CT-BHI (*n* = 258)	Intermediate CT-BHI (*n* = 256)	Higher CT-BHI (*n* = 253)	*P* value
CACS					
Median (interquartile range)	54 (0–352)	146 (4–548)	39 (0–269)	25 (0–277)	*p* < 0.001
CACS 0	245 (32)	59 (23)	92 (36)	94 (37)	*p* = 0.003
CACS 1–99	187 (24)	61 (24)	64 (25)	62 (25)	
CACS 100–399	161 (21)	63 (24)	48 (19)	50 (20)	
CACS ≥ 400	174 (23)	75 (29)	52 (20)	47 (19)	
CACS ≥ 140	307 (40)	131 (51)	87 (34)	89 (35)	*p* < 0.001
CCTA					
CAD-RADS 0	199 (26)	45 (17)	76 (30)	78 (31)	*p* = 0.037
CAD-RADS 1–2	286 (37)	106 (41)	92 (36)	88 (35)	
CAD-RADS 3	134 (18)	46 (19)	43 (17)	45 (18)	
CAD-RADS 4A	86 (11)	39 (15)	22 (9)	25 (10)	
CAD-RADS 4B	14 (2)	5 (2)	6 (2)	3 (1)	
CAD-RADS 5	48 (6)	17 (7)	17 (7)	14 (6)	

Values are n (%), if not indicated otherwise

*CACS* coronary artery calcium score, *CCTA* coronary computed tomography angiography, *CAD-RADS* Coronary Artery Disease Reporting and Data System

Regarding radiation dose, the mean dose-length products of CAC scoring and CCTA were 16 ± 9 and 219 ± 249 mGy·cm, respectively.

### Correlation between CT-BHI and DXA indices

Lumbar spine DXA data obtained within 1 year of CAC scoring were available in 44 patients (Supplemental Table 1). In this validation subset, CT-BHI showed significant positive correlations with T-score (Supplemental Table 2; Spearman *ρ* = 0.509; *p* < 0.001) and BMD (Spearman *ρ* = 0.503; *p* < 0.001). After adjustment for age, sex, body mass index, and the interval between CAC scoring and DXA, each 10-HU increase in CT-BHI remained significantly associated with higher T-score (Supplemental Table 3; β = 0.208; 95% CI, 0.091–0.325; *p* = 0.001) and higher BMD (β = 0.025 g/cm^2^; 95% CI, 0.011–0.039; *p* = 0.001). Across CT-BHI tertiles, T-score and BMD increased in a graded manner, with significant between-group differences (Supplemental Table 4).

### Survival analysis

During a median follow-up of 5.8 years, 63 events occurred. In ROC curve analyses for all-cause mortality, the areas under the curve for CT-BHI and CACS were 0.60 and 0.64, respectively. The exploratory optimal cutoff values determined by the Youden index were 155 HU for CT-BHI and 140 for CACS. The bootstrap-derived 95% confidence intervals for the CT-BHI and CACS cutoffs were 103–182 HU and 86–241, respectively. These ROC-derived cutoffs were used for the subsequent Kaplan–Meier survival analyses and Cox regression models.

**Fig. 2:**
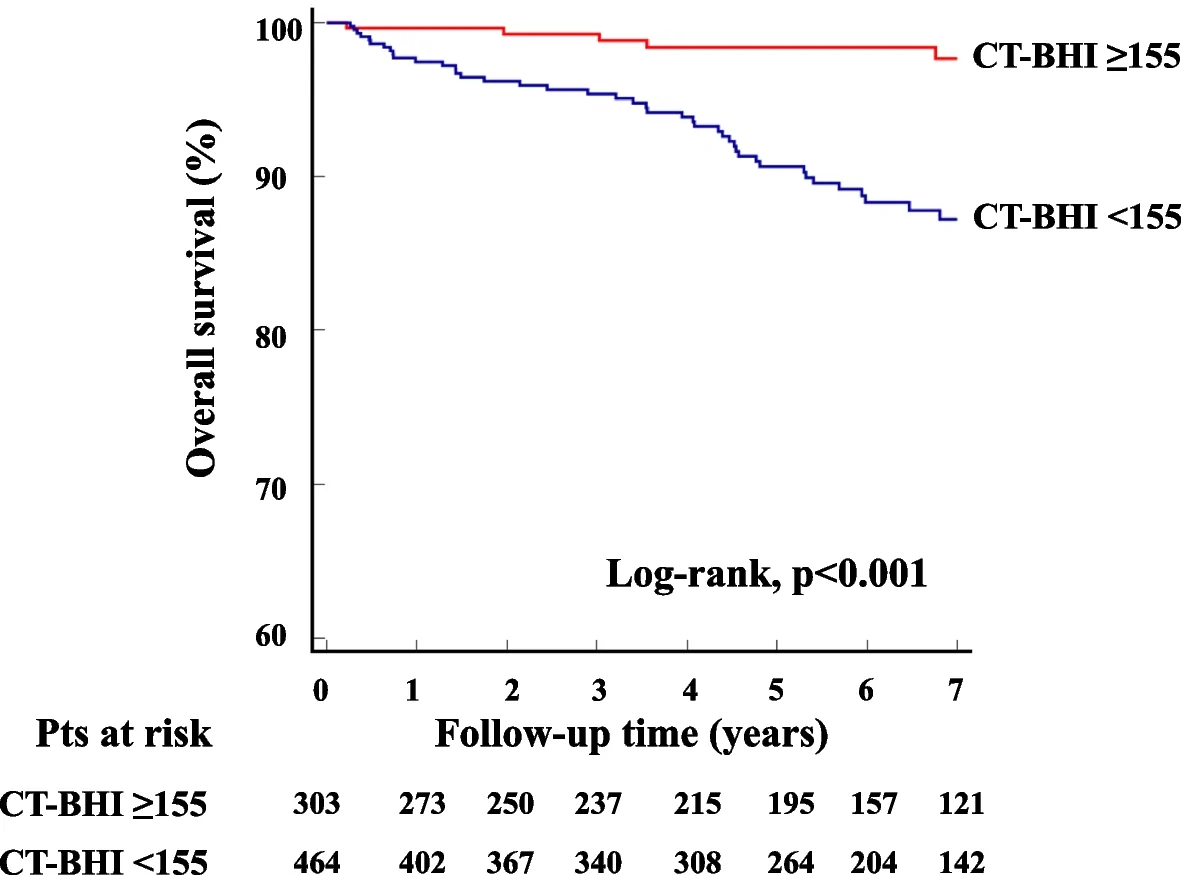
Kaplan–Meier survival curves for all-cause mortality stratified by CT-BHI. The cumulative overall survival rate was significantly lower in patients with lower CT-BHI (< 155 HU) compared to those with higher CT-BHI (≥ 155 HU) (log-rank test, *p* < 0.001). CT-BHI, computed tomography-based bone health indicator; HU, Hounsfield unit

**Fig. 3:**
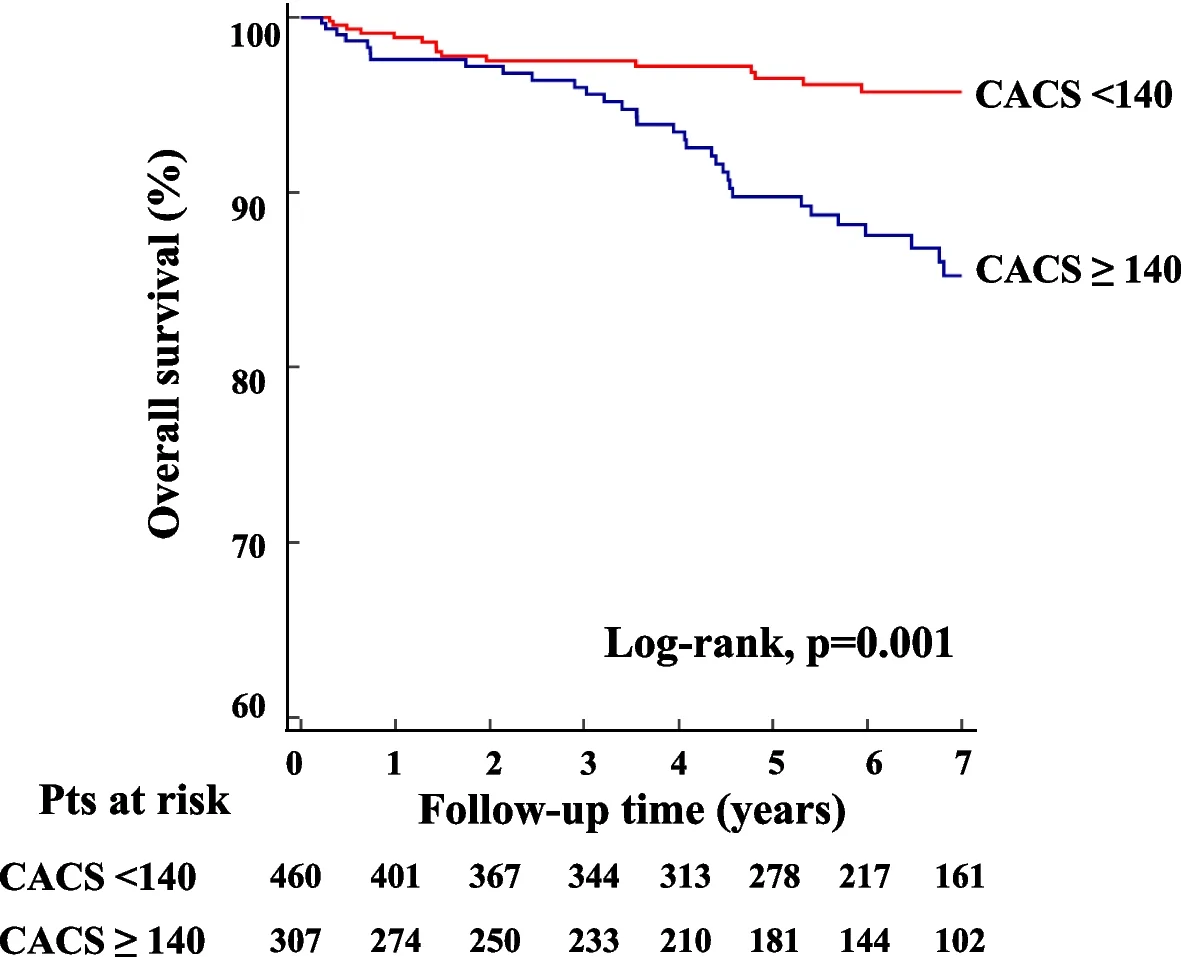
Kaplan–Meier survival curves for all-cause mortality stratified by CACS. Patients with a higher CACS (≥ 140) demonstrated significantly poorer overall survival compared to those with a lower CACS (< 140) (log-rank test, *p* = 0.001). CACS, coronary artery calcium score

Figure 2 illustrates the Kaplan–Meier curves for all-cause mortality, stratifying the cohort by the CT-BHI cutoff value of 155. Patients (*n* = 464) in the CT-BHI < 155 group exhibited significantly lower overall survival compared to those (*n* = 303) in the CT-BHI ≥ 155 group (log-rank, *p* < 0.001). As shown in Fig. 3, patients (*n* = 307) with a higher CACS (≥ 140) had significantly lower overall survival rates compared to those (*n* = 460) with a lower CACS (< 140) (log-rank, *p* = 0.001).

**Fig. 4:**
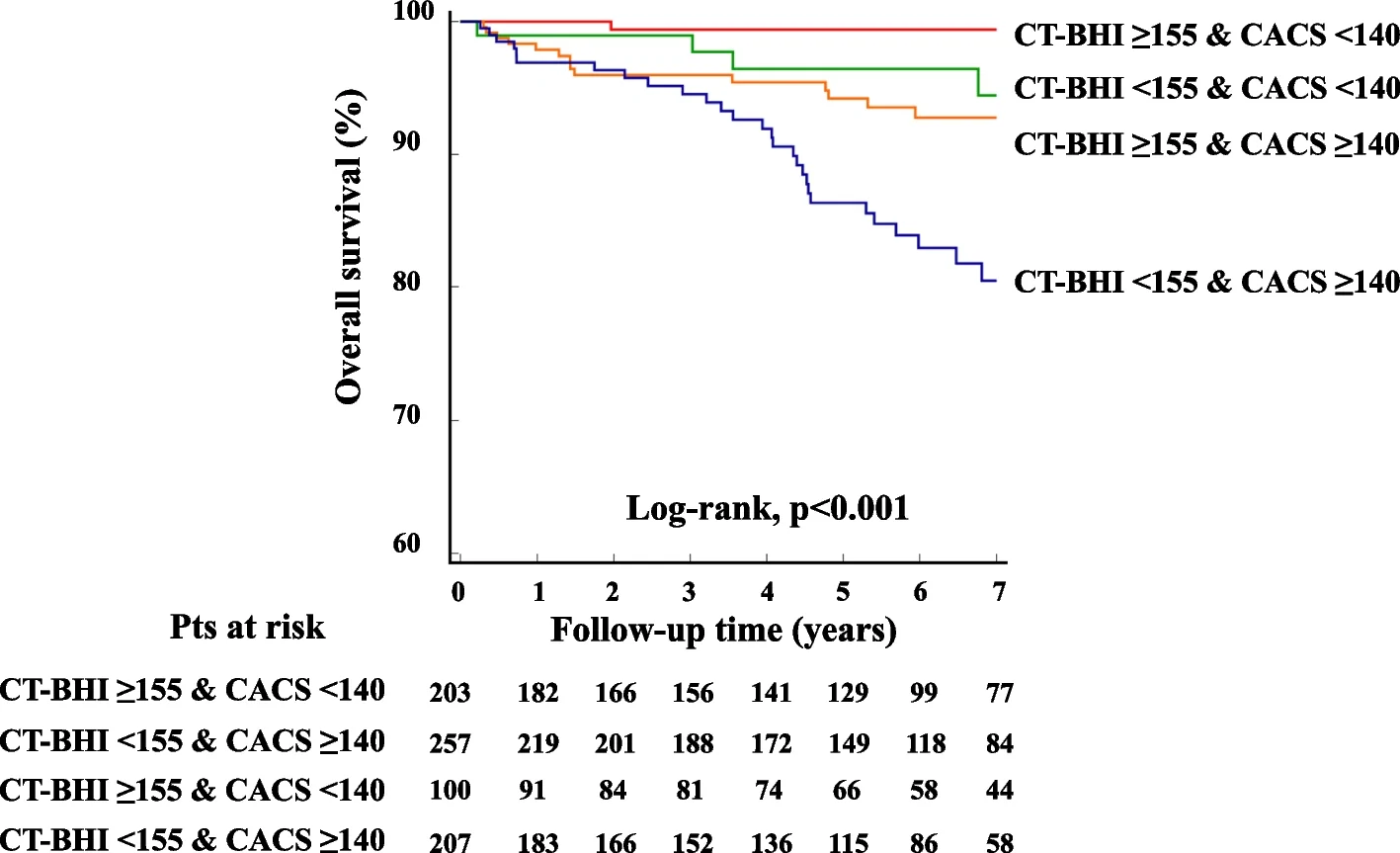
Kaplan–Meier survival curves for all-cause mortality stratified by the combination of CT-BHI and CACS. Patients were categorized into four groups based on CT-BHI (cutoff, 155 HU) and CACS (cutoff, 140). The Kaplan–Meier analysis reveals a distinct graded association between CT-BHI and coronary calcification and all-cause mortality. The group with concomitant lower CT-BHI (< 155 HU) and higher CACS (≥ 140) exhibited the lowest survival rate among the four groups (log-rank test, *p* < 0.001). CACS, coronary artery calcium score; CT-BHI, computed tomography-based bone health indicator; HU, Hounsfield unit

Figure 4 shows the Kaplan–Meier survival analysis stratified by a combination of both the CT-BHI and the CACS. The cohort was divided into four distinct risk groups: CT-BHI ≥ 155 & CACS < 140 (*n* = 203), CT-BHI < 155 & CACS < 140 (*n* = 257), CT-BHI ≥ 155 & CACS ≥ 140 (*n* = 100), and CT-BHI < 155 & CACS ≥ 140 (*n* = 207). The analysis demonstrated a clear risk stratification among the groups. Patients with both CT-BHI < 155 and CACS ≥ 140 had the worst overall survival. A significant difference in survival was observed among the four groups (log-rank, *p* < 0.001).

Table 3 summarizes the results of the univariate and multivariable Cox proportional hazards regression analyses for all-cause mortality. The univariate analysis showed that CACS ≥ 140 (HR 3.1, 95%CI 1.9–5.3; *p* < 0.001), obstructive CAD on CCTA (HR 1.8, 95%CI 1.1–3.0; *p* = 0.018), and CT-BHI < 155 (HR 2.7, 95%CI 1.5–4.8; *p* = 0.001) were all significant predictors. To assess the independent prognostic value of CT-BHI while avoiding multicollinearity between coronary calcification and stenosis, two separate multivariable models were constructed. In Model 1, which included both CACS and CT-BHI, a CACS ≥ 140 (HR 2.9, 95%CI 1.7–5.0; *p* < 0.001) and CT-BHI < 155 (HR 2.4, 95%CI 1.3–4.4; *p* = 0.003) were both significant independent predictors of mortality. In Model 2, which included CCTA findings and CT-BHI, both obstructive CAD (HR 1.7, 95%CI 1.1–2.8; *p* = 0.031) and CT-BHI < 155 (HR 2.6, 95%CI 1.4–4.7; *p* = 0.002) remained independent predictors.

**Table 3:** Cox proportional hazards regression analysis

Parameters	Univariate		Multivariate model 1		Multivariate model 2	
	HR (95%CI)	*p* value	HR (95%CI)	*p* value	HR (95%CI)	*p* value
CACS ≥ 140	3.1 (1.9–5.3)	< 0.001	2.9 (1.7–5.0)	< 0.001		
Obstructive CAD	1.8 (1.1–3.0)	0.018			1.7 (1.1–2.8)	0.031
CT-BHI < 155	2.7 (1.5–4.8)	0.001	2.4 (1.3–4.4)	0.003	2.6 (1.4–4.7)	0.002

*CI* confidence interval, *CACS* coronary artery calcium score, *CAD* coronary artery disease, *CT-BHI* computed tomography-based bone health indicator, *HR* hazard ratio

Adding CT-BHI to CACS significantly yielded a continuous NRI of 0.263 (*p* = 0.041) and an IDI of 0.0080 (*p* = 0.021), which indicates the incremental prognostic utility of CT-BHI beyond the information provided by CACS.

#### Renal function sensitivity analyses

Renal function according to CT-BHI tertiles is shown in Supplemental Table 5. Median eGFR tended to be lower in the lower CT-BHI group than in the intermediate and higher CT-BHI groups, although the difference did not reach statistical significance (*p* = 0.076). Serum creatinine levels were similar across CT-BHI tertiles, whereas the prevalence of eGFR < 60 mL/min/1.73 m^2^ was significantly higher in the lower CT-BHI group than in the intermediate and higher groups (30.6%, 22.7%, and 22.1%, respectively; *p* = 0.046).

In Cox regression sensitivity analyses, additional adjustment for eGFR or creatinine did not materially change the prognostic value of CT-BHI (Supplemental Table 6). In the model including CT-BHI, CACS, and eGFR, CT-BHI < 155 HU remained independently associated with all-cause mortality (HR, 2.44; 95% CI, 1.35–4.44; *p* = 0.003). Similarly, in the model including CT-BHI, obstructive CAD, and eGFR, CT-BHI < 155 HU remained significant (HR, 2.55; 95% CI, 1.41–4.64; *p* = 0.002). Consistent results were obtained when creatinine was added instead of eGFR.

## Discussion

The principal finding of this study is that lower CT-BHI, an opportunistic bone health marker for osteoporosis derived from CAC scans, is significantly associated with a higher burden of coronary artery calcification. Furthermore, we demonstrated that a lower CT-BHI is an independent predictor of all-cause mortality, even after adjusting for CACS. The combination of both CT-BHI and CACS enhanced risk stratification, suggesting that these two markers provide additive and complementary prognostic information.

The finding of an inverse relationship between CT-BHI and CACS is consistent with previous studies that have reported a link between low BMD and vascular calcification [5, 7–9]. For instance, in the Multiethnic Study of Atherosclerosis (MESA), the prevalence of any CAC (CACS > 0) was significantly higher in individuals with osteoporosis (68%) compared to those with normal BMD (36%) [5]. Similarly, another study reported that the mean CACS in patients with osteoporosis at the femoral neck was nearly three times higher than in those with normal BMD (126.9 vs. 43.2, respectively) [7]. The underlying mechanisms for this association, often referred to as the “calcification paradox,” are complex and not fully elucidated, but are thought to involve shared pathophysiological pathways. These include chronic inflammation, the RANK/RANKL/OPG signaling pathway, oxidative stress, and deficiencies in vitamins D and K, all of which play crucial roles in both bone metabolism and arterial calcification [11, 12]. On the other hand, some studies have failed to show significant difference in CACS between groups with and without osteoporosis [13]. These discrepancies might be attributable to differences in study populations, BMD measurement sites, and methodologies. Our study used CT attenuation values, which may be less susceptible to confounders like degenerative spinal changes and aortic calcification that can artificially inflate BMD values measured by DXA [6]. This methodological advantage may have contributed to the clear association observed in this cohort.

The independent prognostic value of low CT-BHI for all-cause mortality supports the findings of previous large cohort studies linking osteoporosis to increased mortality. Tankó et al. demonstrated that postmenopausal women with osteoporosis had a 3.9-fold increased risk for cardiovascular events compared with women with low bone mass (95% CI, 2.0–7.7) [3]. In another study, each standard deviation decrease in bone mineral content in early postmenopausal women was associated with a 2.3-fold increase in the relative risk of dying from cardiovascular disease within 17 years (95% CI, 1.0 to 5.3) [4]. What is particularly significant in our study is that CT-BHI remained a robust predictor even after accounting for the CACS, which is one of the most powerful predictors of future events. This suggests that while osteoporosis and atherosclerosis may be intertwined, they also likely contribute to adverse outcomes through partially independent pathways. The mortality risk associated with low bone mass may reflect not only its link to vascular disease but also to overall frailty, malnutrition, and reduced physical activity, which are themselves powerful predictors of mortality.

Renal dysfunction may affect both vascular calcification and bone metabolism through mechanisms related to chronic kidney disease-mineral and bone disorder (CKD-MBD). In the present study, eGFR tended to be reduced in patients with lower CT-BHI; however, additional adjustment for eGFR or creatinine did not attenuate the association between low CT-BHI and all-cause mortality. These findings suggest that the prognostic value of CT-BHI was not explained solely by routinely measured renal function. Nevertheless, because detailed CKD-MBD markers were not available, residual confounding by renal bone-mineral metabolism cannot be excluded.

Our results extend the body of previous research that has established a link between thoracic bone mineral density, coronary atherosclerosis, and long-term prognosis. Studies by Chen et al. [14] and Ahmadi et al. [15] demonstrated that lower vertebral attenuation is associated with increased CAC burden and mortality risk. Those studies focused on restricted populations, specifically end-stage renal disease patients or asymptomatic cohorts, and Ahmadi et al. relied on calibration phantoms for quantitative bone density assessment. In contrast, our approach used routine non-contrast CT attenuation values in a broad clinical population of patients with suspected CAD. This demonstrates the utility of a purely opportunistic screening method without the need for additional equipment or costs. More importantly, unlike prior research that was limited to CAC scoring, we incorporated CCTA data to evaluate luminal stenosis. The current study demonstrated a significant inverse association between thoracic vertebral attenuation and CAD severity based on the CAD-RADS classification. This comprehensive assessment enabled us to establish that lower vertebral attenuation remains an independent predictor of all-cause mortality even after adjusting for the presence of obstructive CAD, thereby offering more robust prognostic value in the management of suspected CAD patients.

### Clinical implications

The findings of this study have practical clinical implications. Given the widespread use of CACS, our results indicate the dual utility of this modality, extending its role from strictly coronary artery evaluation to the simultaneous screening of bone health. This approach can be seamlessly integrated into the existing clinical workflow, providing crucial insights into two distinct yet interconnected disease processes without any additional cost or radiation exposure to the patient. Since osteoporosis is often silent and frequently goes undiagnosed until a fragility fracture occurs, this opportunistic screening offers a vital window to identify at-risk individuals who would otherwise go unnoticed.

The integrated assessment of both CACS and CT-BHI could lead to a more comprehensive and accurate patient risk profile than either marker provides alone. For example, a patient with a moderate CACS, who might not be considered for the most aggressive cardiovascular prevention, could be re-stratified into a higher overall risk category if their CT-BHI is found to be very low. This understanding can directly influence clinical management, justifying more intensive preventive strategies targeting both cardiovascular and skeletal health. To make this information actionable, radiology reports for CACS could be standardized to include a comment on the vertebral attenuation, thereby alerting the referring physician to the potential need for further bone health investigation.

### Limitations

Several limitations of our study should be acknowledged. First, this was a retrospective, single-center study, which may limit the generalizability of our findings. Second, although we performed an exploratory validation analysis using available DXA data, DXA was available only in a limited subset of patients and was not systematically obtained in the entire cohort. Therefore, CT-BHI should not be interpreted as a diagnostic substitute for DXA-defined osteoporosis. Third, our primary outcome was all-cause mortality; cause-specific mortality data, particularly cardiovascular mortality, were not available, which would have provided deeper insight into the observed association. Fourth, the CT-BHI cutoff of 155 HU used for survival analyses was derived from the present cohort and should be considered exploratory. External validation is required to determine whether this threshold is generalizable to other populations and CT acquisition settings. Fifth, quantitative CT (QCT)-based validation would provide a more direct quantitative assessment because QCT can estimate volumetric BMD in mg/cm^3^. In contrast, CT-BHI is derived from uncalibrated vertebral CT attenuation in HU and should be regarded as an opportunistic CT-based bone health indicator rather than a direct measure of volumetric BMD. Further studies using phantom-based or validated phantomless QCT are warranted to establish quantitative BMD correlates and clinically applicable thresholds. Sixth, although CAC images were reviewed before CT-BHI measurement, subtle degenerative or endplate-related changes may still have influenced vertebral attenuation measurements. Modic changes were not systematically assessed because magnetic resonance imaging data were not available. Finally, although we performed additional sensitivity analyses using available eGFR and serum creatinine data, renal function was assessed only using these routinely available indices. Detailed parameters related to CKD-MBD, including serum calcium, phosphate, parathyroid hormone, and vitamin D levels, were not systematically available. In addition, information on several potential confounders related to bone metabolism, nutritional status, and frailty, including glucocorticoid use, bisphosphonate use, hormone replacement therapy, thiazolidinedione use, serum albumin, physical activity, and formal frailty indices, was not sufficiently available for incorporation into the multivariable models. These unmeasured factors may influence both vertebral CT attenuation and mortality risk.

## Conclusions

Lower CT-BHI, measured opportunistically from CAC scans, was significantly associated with the presence of CAD and is an independent predictor of all-cause mortality. The integrated assessment of both bone health and coronary calcification from a single CT scan provides a more comprehensive risk assessment than either marker alone.

## Supplementary Information

Supplementary Material 1.

Supplementary Material 2.

## Data Availability

The datasets generated and/or analysed during the current study are not publicly available due to patient privacy and institutional restrictions but are available from the corresponding author on reasonable request.

## Abbreviations

BMD: Bone mineral density
CAC: Coronary artery calcium
CACS: Coronary artery calcium score
CAD: Coronary artery disease
CAD-RADS: Coronary artery disease reporting and data system
CKD-MBD: Chronic kidney disease-mineral and bone disorder
CCTA: Coronary computed tomography angiography
CI: Confidence interval
CT: Computed tomography
CT-BHI: Computed tomography-based bone health indicator
DXA: Dual-energy X-ray absorptiometry
ECG: Electrocardiography/electrocardiographic
eGFR: Estimated glomerular filtration rate
HR: Hazard ratio
IQR: Interquartile range
MESA: Multiethnic Study of Atherosclerosis
QCT: Quantitative computed tomography
ROI: Region of interest
SD: Standard deviation

## References

[1] Global Burden of Cardiovascular Diseases and Risks 2023 Collaborators. Global, regional, and national burden of cardiovascular diseases and risk factors in 204 countries and territories, 1990-2023. J Am Coll Cardiol. 2025;86:2167–243. . 10.1016/j.jacc.2025.08.015 40990886

[2] Cummings SR, Melton LJ. Epidemiology and outcomes of osteoporotic fractures. Lancet. 2002;359:1761–7. 10.1016/S0140-6736(02)08657-9. 12049882

[3] Tankó LB, Christiansen C, Cox DA, Geiger MJ, McNabb MA, Cummings SR. Relationship between osteoporosis and cardiovascular disease in postmenopausal women. J Bone Miner Res. 2005;20:1912–20. 10.1359/JBMR.050711. 16234963

[4] von der Recke P, Hansen MA, Hassager C. The association between low bone mass at the menopause and cardiovascular mortality. Am J Med. 1999;106:273–8. 10.1016/S0002-9343(99)00028-5. 10190374

[5] Manubolu VS, Mao S, Kinninger A, Dahal S, Ahmad K, Havistin R, et al. Association between coronary artery calcium and thoracic spine bone mineral density: multiethnic study of atherosclerosis (MESA). Nutr Metab Cardiovasc Dis. 2023;33:532–40. 10.1016/j.numecd.2022.12.006. PMC9974807 36642601

[6] Therkildsen J, Nissen L, Jørgensen HS, et al. Thoracic bone mineral density derived from cardiac CT is associated with greater fracture rate. Radiology. 2020;296:499–508. 10.1148/radiol.2020192706. 32662758

[7] Xu R, Yang HN, Li YQ, Wang QF, Guo AH, Ayiti A, et al. Association of coronary artery calcium with bone mineral density in postmenopausal women. Coron Artery Dis. 2016;27:586–91. 10.1097/MCA.0000000000000402. 27363000

[8] Lin T, Liu JC, Chang LY, Shen CW. Association between coronary artery calcification using low-dose MDCT coronary angiography and bone mineral density in middle-aged men and women. Osteoporos Int. 2011;22:627–34. 10.1007/s00198-010-1303-5. 20552331

[9] Akin MN, Altun I. Associations of coronary plaque characteristics and coronary calcification with bone mineral density in postmenopausal women. Eur Rev Med Pharmacol Sci. 2022;26:7616–22. 10.26355/eurrev_202210_30037. 36314333

[10] Cury RC, Leipsic J, Abbara S, Achenbach S, Berman D, Bittencourt M, et al. CAD-RADS™ 2.0 - 2022 coronary artery disease-reporting and data system: an expert consensus document of the Society of Cardiovascular Computed Tomography (SCCT), the American College of Cardiology (ACC), the American College of Radiology (ACR), and the North America Society of Cardiovascular Imaging (NASCI). J Cardiovasc Comput Tomogr. 2022;16:536–57. 10.1016/j.jcct.2022.07.002. 35864070

[11] Frost ML, Grella R, Millasseau SC, Jiang BY, Hampson G, Fogelman I, et al. Relationship of calcification of atherosclerotic plaque and arterial stiffness to bone mineral density and osteoprotegerin in postmenopausal women referred for osteoporosis screening. Calcif Tissue Int. 2008;83:112–20. 10.1007/s00223-008-9153-2. 18612580

[12] Bandeira F, de Oliveira LB, Caldeira RB, Toscano LS. Skeletal consequences of heart failure. Womens Health (Lond). 2022;18:17455057221135501. 10.1177/17455057221135501. PMC9634191 36321835

[13] Zhang P, Yang L, Xu Q, et al. Associations between bone mineral density and coronary artery calcification: a systematic review and meta-analysis. Ther Adv Chronic Dis. 2022;13:20406223221086998. 10.1177/20406223221086998. PMC8972925 35371431

[14] Chen Z, Qureshi AR, Ripsweden J, Wennberg L, Heimburger O, Lindholm B, et al. Vertebral bone density associates with coronary artery calcification and is an independent predictor of poor outcome in end-stage renal disease patients. Bone. 2016;92:50–7. 10.1016/j.bone.2016.08.007. 27519971

[15] Ahmadi N, Mao SS, Hajsadeghi F, Arnold B, Kiramijyan S, Gao Y, et al. The relation of low levels of bone mineral density with coronary artery calcium and mortality. Osteoporos Int. 2018;29:1609–16. 10.1007/s00198-018-4524-7. 29704026

