# Supplementary material for: Thoracic vertebral CT-based bone health indicator from coronary calcium scoring: correlation with CAD and prognostic impact
Source: BMC Cardiovasc Disord. 2026 Jul 16;26:901. doi: 10.1186/s12872-026-06284-9 (PMC13656631; doi:10.1186/s12872-026-06284-9)
Supplement: Supplementary file 1 [file 12872_2026_6284_MOESM1_ESM.docx]

**Supplemental Figure 1. Study flowchart.**

Flowchart of patient selection for the study. A total of 1,904 patients who underwent coronary artery calcium (CAC) scoring and coronary CT angiography (CCTA) for evaluation of coronary artery disease between April 2012 and March 2019 were initially screened. After exclusion of patients with non-ischemic cardiomyopathy, prior myocardial infarction, prior coronary revascularization, severe valvular disease, aortic disease, congenital heart disease, cardiac tumors, or CAC scans unsuitable for CT-BHI assessment (cases with severe vertebral deformities or lower thoracic vertebrae outside the field of view), 767 patients were included in the final study cohort.

CT-BHI, computed tomography-based bone health indicator.

**Supplemental Table 1. Lumbar spine DXA validation subset**

| **Characteristic** | **Value** |
| --- | --- |
| Patients with lumbar spine DXA within 1 year | 44 |
| Age, years | 71.0 [64.8, 73.0] |
| Male sex | 7 (15.9%) |
| Body mass index, kg/m^2^ | 19.7 [17.3, 21.5] |
| CT-DXA interval, days | 108 [55, 181] |
| CT-BHI, HU | 112.4 [77.1, 145.8] |
| T-score | -1.25 [-1.92, 0.00] |
| BMD, g/cm^2^ | 0.973 [0.894, 1.138] |

Values are median [interquartile range] or n (%). BMD, Bone mineral density; CT-BHI, Computed tomography-bone health indicator; DXA, Dual Energy X-ray absorptiometry.

**Supplemental Table 2. Correlation between CT-BHI and lumbar spine DXA indices**

| **Lumbar spine DXA index** | **Spearman ρ** | **P value** |
| --- | --- | --- |
| T-score | 0.509 | <0.001 |
| BMD, g/cm^2^ | 0.503 | <0.001 |

CT-BHI, computed tomography-bone health indicator; DXA, Dual Energy X-ray absorptiometry.

**Supplemental Table 3. Linear regression analysis**

| **Lumbar spine DXA index** | **Unadjusted beta per 10 HU (95% CI)** | **p** | **Adjusted beta per 10 HU (95% CI)** | **p** |
| --- | --- | --- | --- | --- |
| T-score | 0.202 (0.091 to 0.314) | <0.001 | 0.208 (0.091 to 0.325) | <0.001 |
| BMD, g/cm^2^ | 0.024 (0.010 to 0.038) | 0.001 | 0.025 (0.011 to 0.039) | <0.001 |

Beta represents the expected change in the DXA index per 10-HU increase in CT-BHI. Adjusted models included age, sex, body mass index, and CT-DXA interval. BMD, Bone mineral density; CT-BHI, Computed tomography-bone health indicator; DXA, Dual Energy X-ray absorptiometry.

**Supplemental Table 4. DXA indices across main-cohort CT-BHI tertiles**

| **Variable** | **Lower CT-BHI** | **Intermediate CT-BHI** | **Higher CT-BHI** | **p value** |
| --- | --- | --- | --- | --- |
| n | 27 | 11 | 6 |  |
| T-score | -1.9 [-2.2, -0.9] | -0.5 [-1.2, 0.9] | 0.8 [-1.2, 1.8] | 0.006 |
| BMD, g/cm^2^ | 0.950 [0.863, 1.009] | 1.056 [0.982, 1.305] | 1.217 [0.971, 1.334] | 0.010 |

Continuous variables are median [interquartile range] and were compared using the Kruskal-Wallis test. BMD, Bone mineral density; CT-BHI, Computed tomography-bone health indicator; DXA, Dual Energy X-ray absorptiometry.

**Supplemental Table 5. Renal function according to CT-BHI tertiles**

| **Variable** | **Lower CT-BHI** | **Intermediate CT-BHI** | **Higher CT-BHI** | **P value** |
| --- | --- | --- | --- | --- |
| eGFR, mL/min/1.73 m² | 69.3 [56.8, 80.8] | 70.7 [61.3, 79.2] | 72.6 [61.2, 82.9] | 0.076 |
| Serum creatinine, mg/dL | 0.75 [0.62, 0.91] | 0.76 [0.65, 0.90] | 0.79 [0.66, 0.91] | 0.455 |
| eGFR <60, n (%) | 79 (30.6) | 58 (22.7) | 56 (22.1) | 0.046 |

Values are median [interquartile range] or n (%). P values were calculated using Kruskal-Wallis tests for continuous variables and chi-square tests for categorical variables.

**Supplemental Table 6. Cox regression sensitivity analyses adjusted for renal function**

| **Model** | **Variable** | **HR (95% CI)** | **p value** |
| --- | --- | --- | --- |
| M1 + eGFR | CT-BHI <155 HU | 2.44 (1.35–4.44) | 0.003 |
| M1 + eGFR | CACS ≥140 | 2.92 (1.72–4.96) | <0.001 |
| M1 + eGFR | eGFR per 10 mL/min/1.73 m² | 0.99 (0.88–1.11) | 0.849 |
| M2 + eGFR | CT-BHI <155 HU | 2.55 (1.41–4.64) | 0.002 |
| M2 + eGFR | Obstructive CAD | 1.70 (1.03–2.80) | 0.038 |
| M2 + eGFR | eGFR per 10 mL/min/1.73 m² | 0.98 (0.87–1.10) | 0.687 |
| M1 + Cr | CT-BHI <155 HU | 2.43 (1.34–4.43) | 0.004 |
| M1 + Cr | CACS ≥140 | 2.97 (1.75–5.05) | <0.001 |
| M1 + Cr | Creatinine per 0.1 mg/dL | 1.00 (0.98–1.01) | 0.714 |
| M2 + Cr | CT-BHI <155 HU | 2.56 (1.41–4.65) | 0.002 |
| M2 + Cr | Obstructive CAD | 1.73 (1.05–2.85) | 0.031 |
| M2 + Cr | Creatinine per 0.1 mg/dL | 1.00 (0.99–1.01) | 0.876 |

M1 includes CT-BHI <155 HU and CACS ≥140; M2 includes CT-BHI <155 HU and obstructive CAD. CACS, coronary artery calcium score; CAD, coronary artery disease; CI, confidence interval; CT-BHI, computed tomography-based bone health indicator; eGFR, estimated glomerular filtration rate; HR, hazard ratio.
