# Supplementary material for: Thoracic vertebral CT-based bone health indicator from coronary calcium scoring: correlation with CAD and prognostic impact
Source: BMC Cardiovasc Disord. 2026 Jul 16;26:901. doi: 10.1186/s12872-026-06284-9 (PMC13656631; doi:10.1186/s12872-026-06284-9)
Supplement: Supplementary file 2 [file 12872_2026_6284_MOESM2_ESM.docx]

**Additional file**

Validation of Vertebral Level Estimation Using the Lung-Liver Interface

To assess the reliability of identifying the Th10 and Th11 vertebrae based on the right lung-liver interface, we performed a validation study in 99 consecutive patients who underwent chest-to-pelvis CT.

Background: This supplementary study aimed to assess the reliability of identifying the Th10 and Th11 vertebrae using the right lung-liver interface as an anatomical landmark.

Methods: We analyzed 99 consecutive patients aged ≥60 years who underwent chest-to-pelvis CT, which allows for definitive vertebral identification. In each scan, the intervertebral disc located closest to the axial plane containing the superior border of the right lung-liver interface was estimated to be the Th10/11 level. The actual vertebral levels were subsequently verified by tracing the spinal column from the cervical spine or sacrum using anatomical landmarks, including the ribs and pelvic bones, as the gold standard.

Results: The estimated landmark correctly corresponded to the actual Th10/11 level in 68.7% (68/99) of patients. Discrepancies were observed as follows: the actual level was Th11/12 (one level caudal) in 22.2% (22/99) and Th9/10 (one level cranial) in 5.1% (5/99) of cases. Larger deviations (≥2 levels) were rare, occurring in only 4.0% of the cohort. Consequently, the estimated vertebral level was within one vertebral level of the actual Th10/11 in 96.0% (95/99) of the cohort. The detailed distribution is shown in the table below.

Conclusion: The right lung-liver interface serves as a reliable anatomical landmark for estimating the lower thoracic vertebral level. Given that 96% of estimations fell within one vertebral level of the true anatomy, this method is considered sufficiently accurate for the opportunistic screening of bone density in cardiac CT scans.

| Actual Vertebral Level | n (%) | Deviation from Estimated Level |
| --- | --- | --- |
| Th10-11 | 68 (69) | 0 (Correct) |
| Th11-12 | 22 (22) | -1 (Caudal) |
| Th9-10 | 5 (5) | +1 (Cranial) |
| Th12-L1 | 2 (2) | -2 (Caudal) |
| Th8-9 | 2 (2) | +2 (Cranial) |
| Total | 99 (100) |  |
